# Research Hotspots and Trends of Endoplasmic Reticulum Stress in Obesity From 2014 to 2024: A CiteSpace Visualization Analysis

**DOI:** 10.7759/cureus.91155

**Published:** 2025-08-28

**Authors:** Xiaotian Zhang, Mengjie Kang, Yuan Cai, Juan jin, Sitong Guo, Yun Liu

**Affiliations:** 1 Pathology and Pathophysiology, Baoji Central Hospital, Baoji, CHN; 2 Pathology and Pathophysiology, Shenyang Medical College, Shenyang, CHN; 3 Clinical Medicine, Yan'an University School of Medicine, Yan'an, CHN; 4 Otolaryngology, Baoji Central Hospital, Baoji, CHN

**Keywords:** citespace, endoplasmic reticulum stress, obesity, unfolded protein response, visual analysis

## Abstract

Over the decade from 2014 to 2024, extensive research has investigated the relationship between endoplasmic reticulum (ER) stress and obesity. Consequently, reviewing and analyzing the emerging trends and focal points in this field is of paramount importance. We retrieved publications on the association between obesity and ER stress from the Web of Science (WoS) Core Collection, limiting the time range to 2014-2024 and restricting the research types to "Articles" and "Reviews" and utilized CiteSpace software (Chen et al., College of Computing and Informatics, Drexel University, Philadelphia, PA) for visual analysis to identify hot topics and emerging trends. A total of 3,847 publications were retrieved, revealing a consistent upward trend in annual publication numbers. The majority of these publications originate from China and the United States. The top three most prolific authors are Jung Tae Woo, Abd El-Aty AM, and Jeong Ji Hoon. Keywords such as "endoplasmic reticulum stress," "insulin resistance," "oxidative stress," "obesity," and "unfolded protein response" have emerged as frequently used terms. These topics have garnered significant attention in recent years. Keyword cluster analysis identified the top three clusters as "follicular dysfunction," "energy homeostasis," and "activating transcription factor." Timeline analysis of keywords indicates that themes such as "energy homeostasis," "palmitic acid-induced stress," "metabolic syndrome," "endothelial cell dysfunction," and "diet-induced nonalcoholic steatohepatitis" span the entire data acquisition period. The analysis of ER stress in obesity from 2014 to 2024 suggests a focus on molecular mechanism analysis, disease association studies, and the development of therapeutic targets. These advancements may lead to revolutionary breakthroughs in the prevention, diagnosis, and treatment of obesity-related diseases.

## Introduction and background

Obesity is a recently defined metabolic syndrome characterized by elevated body weight, high blood lipid levels, and various other features. Obesity is a newly recognized metabolic syndrome characterized by excessive body weight and elevated blood lipid levels. Currently, the prevalence of obesity among adults over 30 years old worldwide has reached 30%. Obesity and its associated complications, such as type 2 diabetes and hypertension, have significantly increased the social and economic burden. It has emerged as a major health threat to humanity and remains a focal point of research in the field of global public health [1].

In individuals with obesity, oxidative stress, ferroptosis, chronic inflammation, and other pathophysiological processes have been shown to be closely linked to endoplasmic reticulum (ER) stress. [2]. ER stress plays a pivotal role in the pathogenesis of obesity-related diseases. Understanding its mechanisms is crucial for developing effective preventive and therapeutic strategies [3]. ER autophagy facilitates the renewal of the rough ER, helping to alleviate the burden of protein folding and mitigate the decline in overall protein synthesis [4]. When misfolded or unfolded proteins accumulate, cells activate the unfolded protein response (UPR) to restore ER homeostasis [5]. Key unfolded protein response (UPR) sensors include IRE1α, ATF6, and PERK, which collaborate with molecular chaperones to promote proper protein folding and decrease new protein synthesis [6]. The persistent accumulation of unfolded proteins can trigger apoptosis by activating apoptotic signaling pathways. ER stress is implicated in the development of various diseases, including neurodegenerative and metabolic disorders [7].

The activation of the UPR in obesity and obesity-related diseases is potentially linked to ER stress within the ER lumen, as well as changes in the ER membrane environment. Obesity-induced ER stress in the liver may result from both extracellular factors, such as lipids, glucose, and cytokines, and intracellular factors, including lipid accumulation within hepatocytes [8]. The chronic activation of the hepatic ER stress response may play a crucial role in the progression from steatosis to non-alcoholic fatty liver disease (NAFLD), resulting in cell death, inflammation, and accelerated metabolic dysfunction [9]. ER stress in the hypothalamus may be a key mechanism contributing to leptin resistance and obesity. Targeting ER stress presents a promising therapeutic strategy to improve leptin sensitivity, boost energy expenditure, and ultimately address obesity [10]. Chronic activation of the UPR is strongly associated with obesity and type 2 diabetes [11]. In obese patients, various factors can activate the UPR pathways, which may have significant implications for the development of obesity-related complications. These factors include increased protein synthesis, the accumulation of mutated or misfolded proteins, inhibition of protein glycosylation, disruptions in ER calcium homeostasis, glucose and energy deprivation, and hypoxia, as well as the presence of pathogens, pathogen-related components, and toxins [12]. Obesity is inherently linked to chronic inflammation, characterized by an excess of key inflammatory mediators, including tumor necrosis factor-alpha (TNF-α) and others [13]. When ER stress cannot be alleviated through the UPR pathway, it activates the IRE1α-dependent apoptotic pathway and leads to PERK phosphorylation, which in turn promotes the production of pro-inflammatory mediators. Moreover, ER stress demonstrates complex interactions with oxidative stress, insulin resistance, and various signaling pathways. Targeting the UPR pathway for the treatment of chronic conditions such as obesity, diabetes, and fatty liver disease is an active area of research [14].

**Figure 1:**
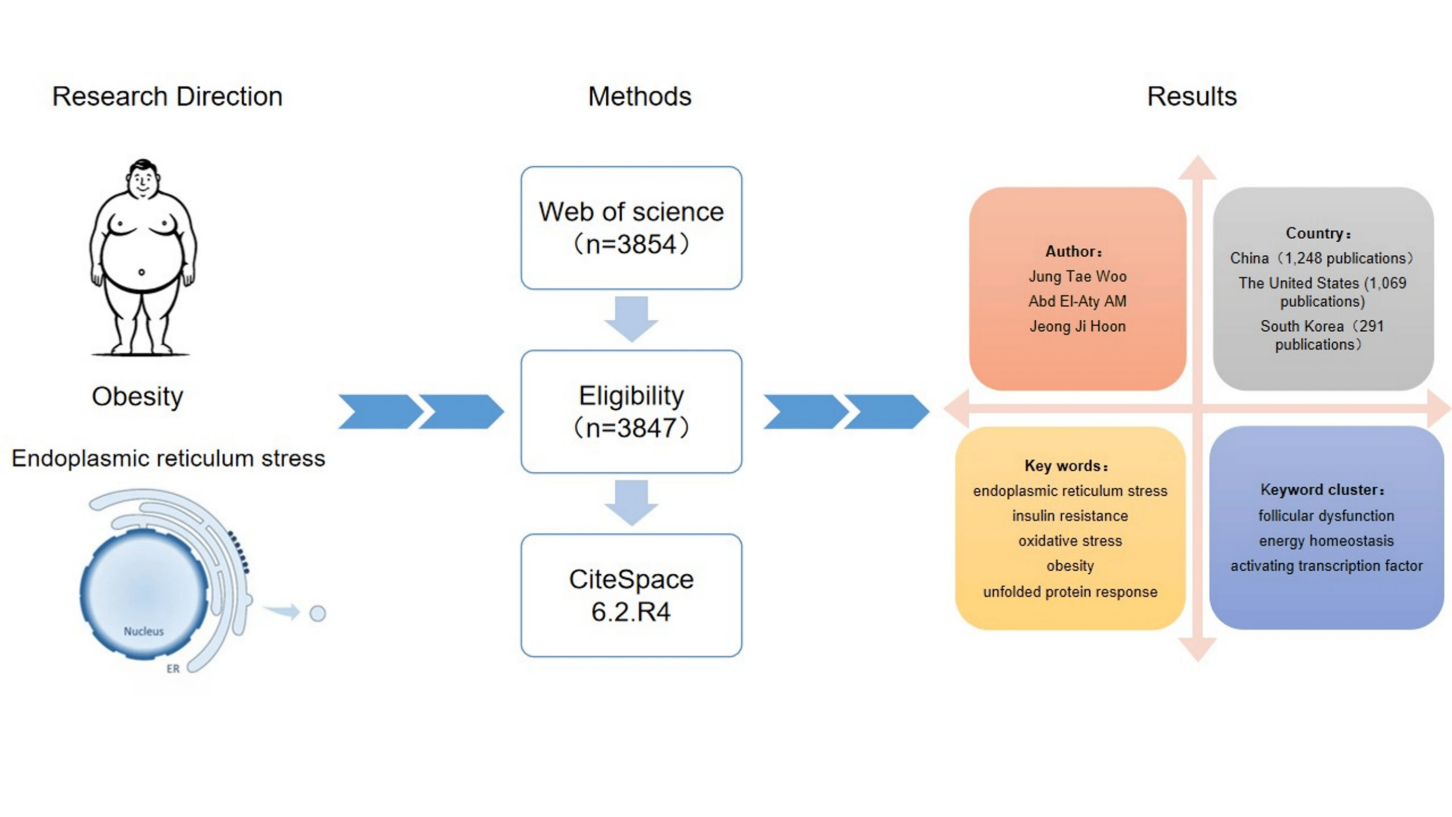
Graphical abstract This figure has been created by the authors.

To the best of our knowledge, there has been no comprehensive academic assessment of the knowledge schema related to obesity and ER stress to date. Consequently, this research aims to utilize CiteSpace visualization (Chen et al., College of Computing and Informatics, Drexel University, Philadelphia, PA) analysis software, leveraging the core database of the Web of Science (WoS), to construct a scientific knowledge map of publications related to obesity and ER stress. The graphical abstract is shown in Figure 1.

## Review

The WoS database mainly collects English literature and covers the major academic research achievements worldwide [15]. Consequently, we conducted our research using the Science Citation Index Expanded within the WoS Core Collection, with the time frame set from 2014 to 2024 and the research type limited to articles and reviews. Boolean operators were employed in the search, and the search formula was as follows: (TS = (obesity OR obesity syndrome OR high-fat diet OR adiposis OR obese)) AND (TS = endoplasmic reticulum stress).

The inclusion criteria were as follows: 1. Publications must have been released between January 1, 2014, and December 31, 2024. 2. The types of research are limited to original articles and review articles. 3. Only documents published in the English language will be considered. The exclusion criteria included 1. Duplicate records; 2. Unpublished or grey literature; 3. Studies whose content does not align with the objectives of this review; 4. Non-relevant document types, including, but not limited to, conference papers, editorials, letters, conference abstracts, book reviews, and corrections. Data collection and processing were conducted independently by two reviewers, with any discrepancies in literature inclusion resolved by a third reviewer. A total of 3,854 papers related to this study were identified, and 3,847 papers remained after manual screening.

CiteSpace is a scientific literature analysis tool developed by Dr. Chen and others [16]. The retrieved documents were converted into data files in TXT format and visualized using CiteSpace 6.2.R4. It is primarily used to analyze the potential knowledge embedded in scientific literature and to present the structure, patterns, and distribution of scientific knowledge through visual means. In the generated visualization model, the size of the circular nodes reflects the frequency or reference count of each node, while the color of the nodes indicates the year in which they were published or referenced. The thickness of the connecting lines between nodes represents the strength of the relationships among items.

**Figure 2:**
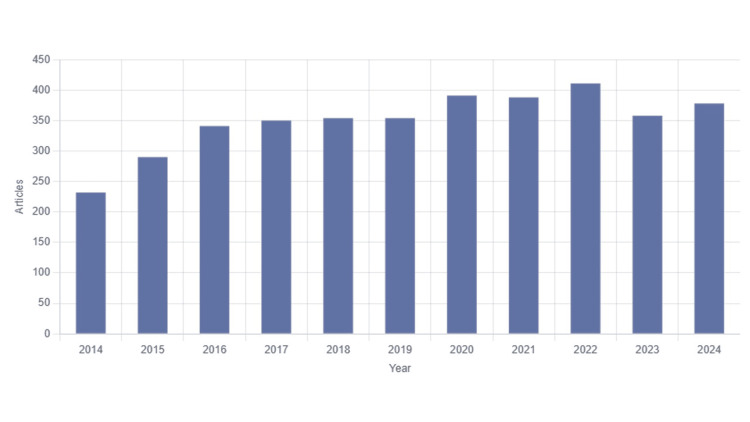
The number of publications in the field of obesity and endoplasmic reticulum stress research from 2014 to 2024.

Figure 2 presents a combination of a bar chart, illustrating the annual trend in the quantity of publications. Between 2014 and 2024, a total of 3,847 publications were retrieved in the field of ER stress in obesity. The overall trend in annual publication numbers reveals a consistent upward trajectory, indicating that research on ER stress in obesity is receiving increasing attention and interest.

**Figure 3:**
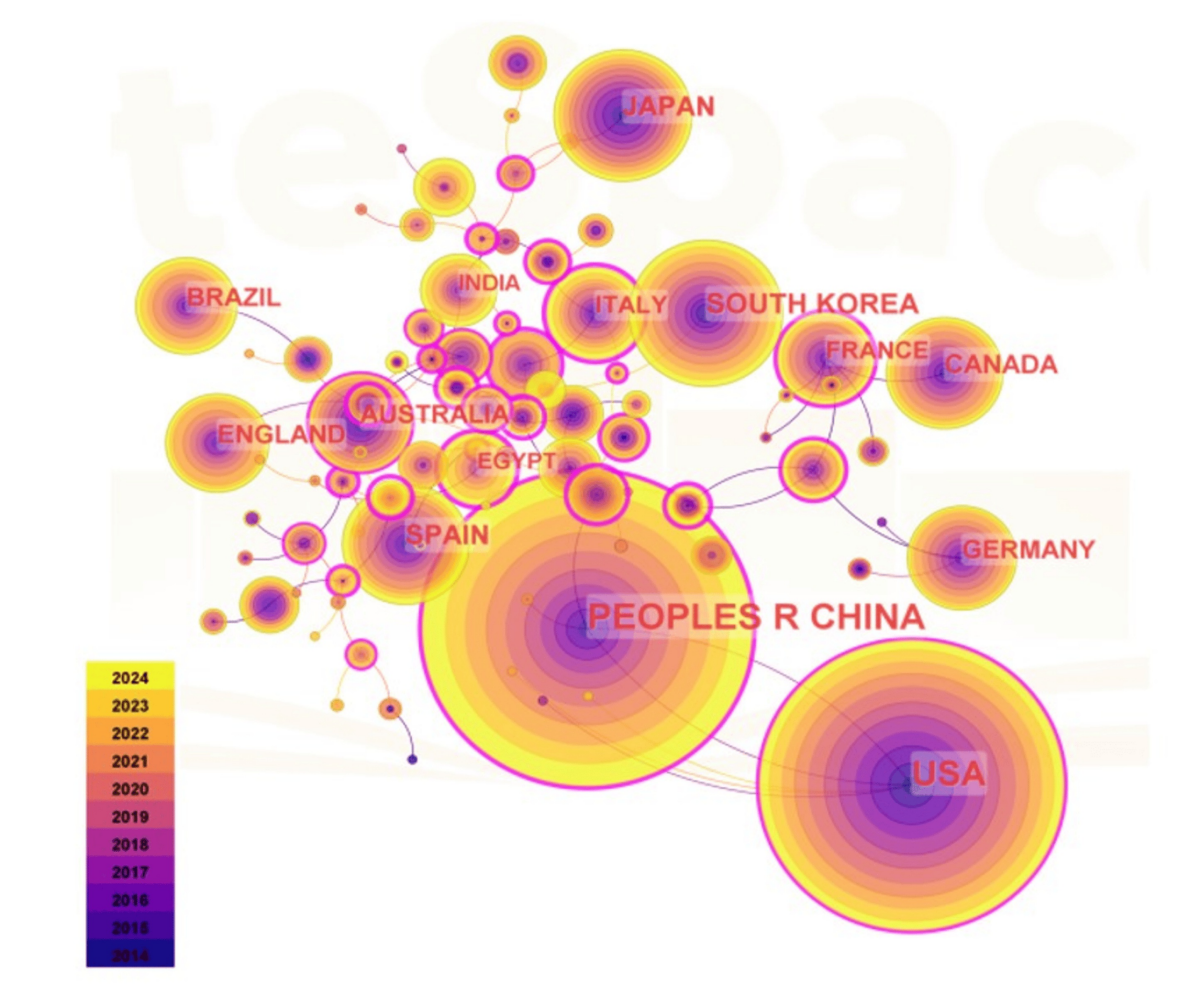
Cooperative analysis in the field of obesity and endoplasmic reticulum stress research among countries/regions The node size represents the co-occurrence frequency. The inner circle represents the older theme, and the outer circle represents the newer theme. The color gradient ranges from purple (2014) to yellow (2024). The connecting lines between nodes represent the occurrence of the collaborative relationship, and the thickness represents the intensity of the collaboration.

Figure 3 illustrates a national network of combined fields, comprising 88 nodes and 96 links. China leads with 1,248 publications, followed by the United States (1,069 publications), South Korea (291 publications), Japan (224 publications), and Spain (209 publications). Notably, only China and the United States exhibit an intermediation centrality exceeding 0.1, underscoring their pivotal roles in obesity and ER stress research. Intermediation centrality serves as an indicator of a country’s or region’s significance within the research network. In this context, the Netherlands ranks highest with a centrality of 0.75, followed by Saudi Arabia and Norway. China leads in the volume of publications, followed by the United States. Notably, despite having fewer publications, the Netherlands, Saudi Arabia, and Norway exhibit the highest intermediary centrality, indicating that these countries may produce higher-quality and more widely recognized research outcomes. 

**Figure 4:**
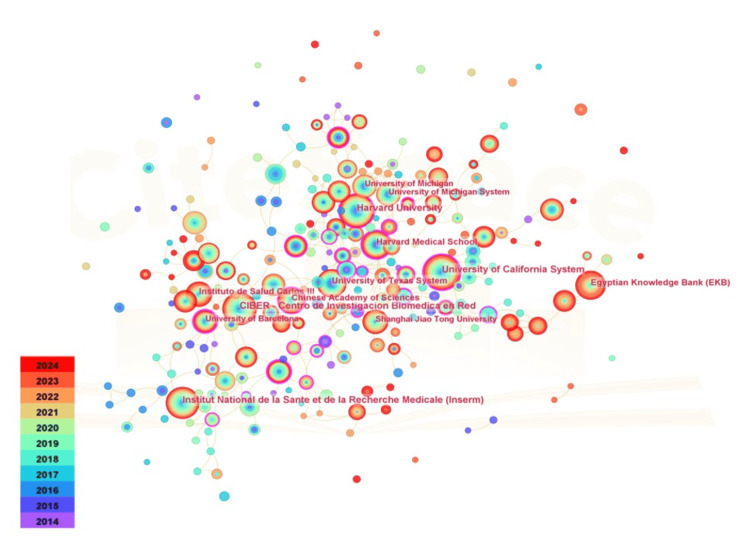
Cooperative analysis among institutions in the field of obesity and endoplasmic reticulum stress research The node size represents the co-occurrence frequency, and the color gradient from purple (2014) to red (2024) indicates the publication year. The connection lines between nodes represent the collaborative relationship, and their thickness indicates the intensity of the collaboration.

Figure 4 presents a co-occurrence analysis of the number of publications by academic institutions. A total of 248 academic institutions have contributed to the literature on obesity and ER stress. The top five institutions, ranked by publication count, are the University of California (106 publications), Centro de Investigación Biomédica en Red (CIBER) (103 publications), Harvard University (102 publications), Institut National de la Santé et de la Recherche Médicale (92 publications), and the Egyptian Knowledge Bank (72 publications). Notably, only the University of California, Harvard University, and Institut National de la Santé et de la Recherche Médicale have an intermediation centrality exceeding 0.1. Harvard University and the University of Michigan share the highest intermediation centrality at 0.16, followed by the University of Texas at 0.15 and the University of California at 0.14, underscoring their pivotal roles in institutional collaboration networks. Among the top five institutions in terms of publication volume, two are from the United States, followed by institutions from Spain, France, and Egypt. However, the top three institutions in terms of intermediary centrality are all located in the United States, underscoring the presence of multiple high-level research institutions in the country and its leadership role in this field.

**Figure 5:**
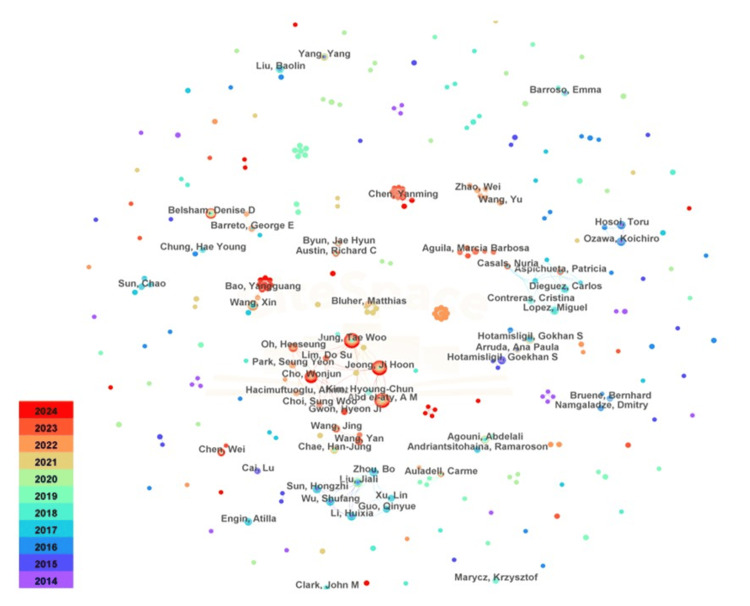
Collaboration among authors Solid nodes represent topics. The innermost circle represents older topics, and the outermost circle represents newer topics. The color gradient range (2014-2024) is as shown in the figure, representing the publication year of the research topic. The thickness of the connecting wire represents the cooperative relationship that occurs.

Figure 5 illustrates the co-occurrence analysis of author publication counts in the fields of obesity and ER stress. Only six authors have published more than 10 papers, with Jung Tae Woo leading the field with 27 publications. He is followed by Abd el-Aty A. M. (24 publications), Jeong Ji Hoon (24 publications), Cho Wonjun (16 publications), Belsham Denise D. (13 publications), and Wang Xin (11 publications). Three authors from Korea, followed by authors from Egypt, Canada, and China. Notably, no author has achieved an intermediary centrality score exceeding 0.1, indicating limited collaboration among researchers in this area.

**Figure 6:**
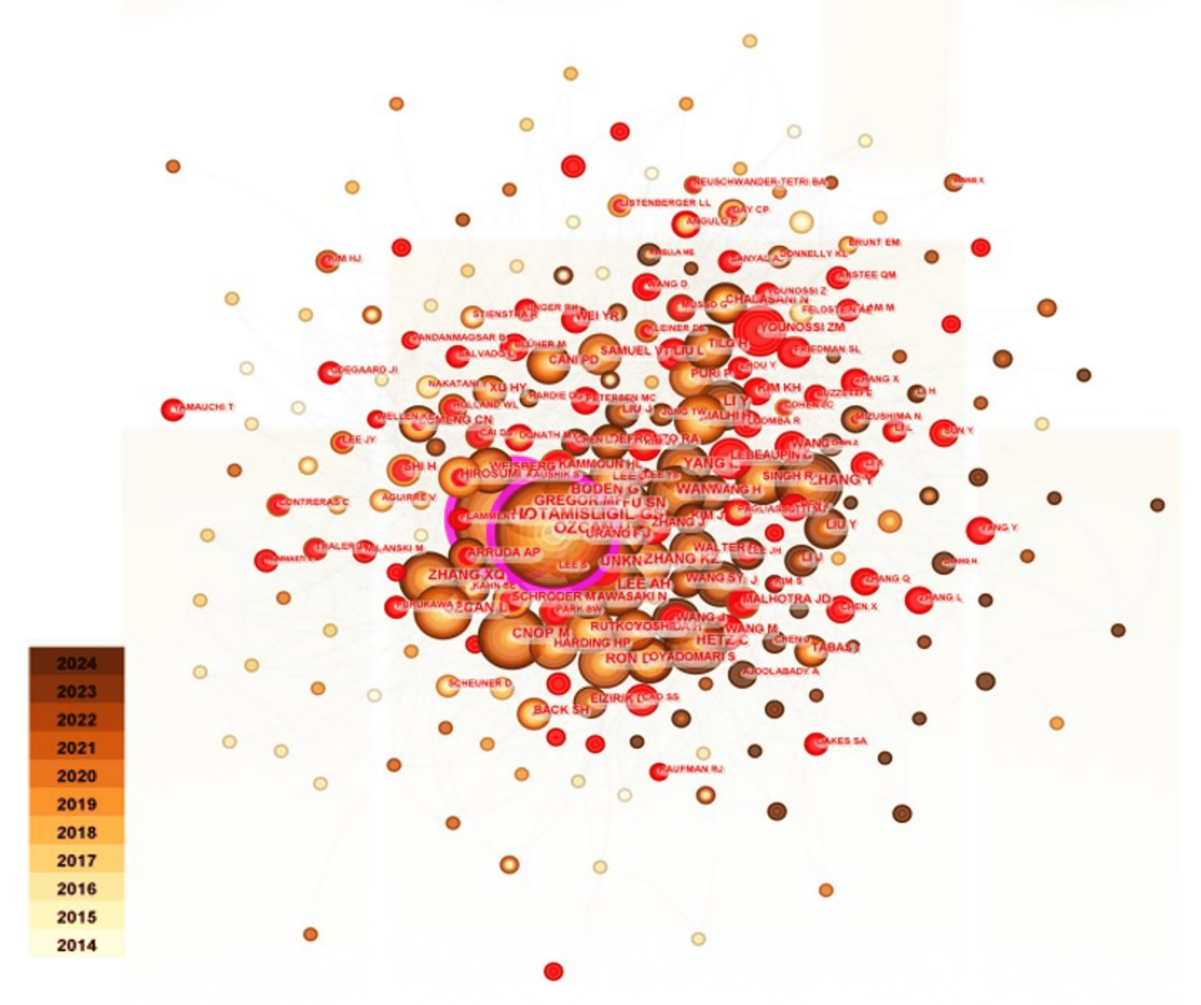
Visual analysis of co-cited authors in the field of obesity and endoplasmic reticulum stress research The node size represents the co-occurrence frequency, and the color gradient from beige (2014) to brown (2024) indicates the publication year. The connection lines between nodes represent the collaborative relationship, and their thickness indicates the intensity of the collaboration.

Figure 6 illustrates the co-citation network as referenced by the authors. The most cited author is Ozcan U, with 893 publications, followed by Hotamisigil GS (838 publications), CnopP M (355 publications), Hetz C (338 publications), and Gregor MF (324 publications). Notably, only three co-cited authors exhibited an intermediation centrality exceeding 0.1. Ozcan U ranked first with a centrality of 0.12, followed by Hotamisigil GS and Samuel VT. This indicates that their contributions to the fields of obesity and ER stress have garnered significant recognition and hold considerable academic influence.

**Figure 7:**
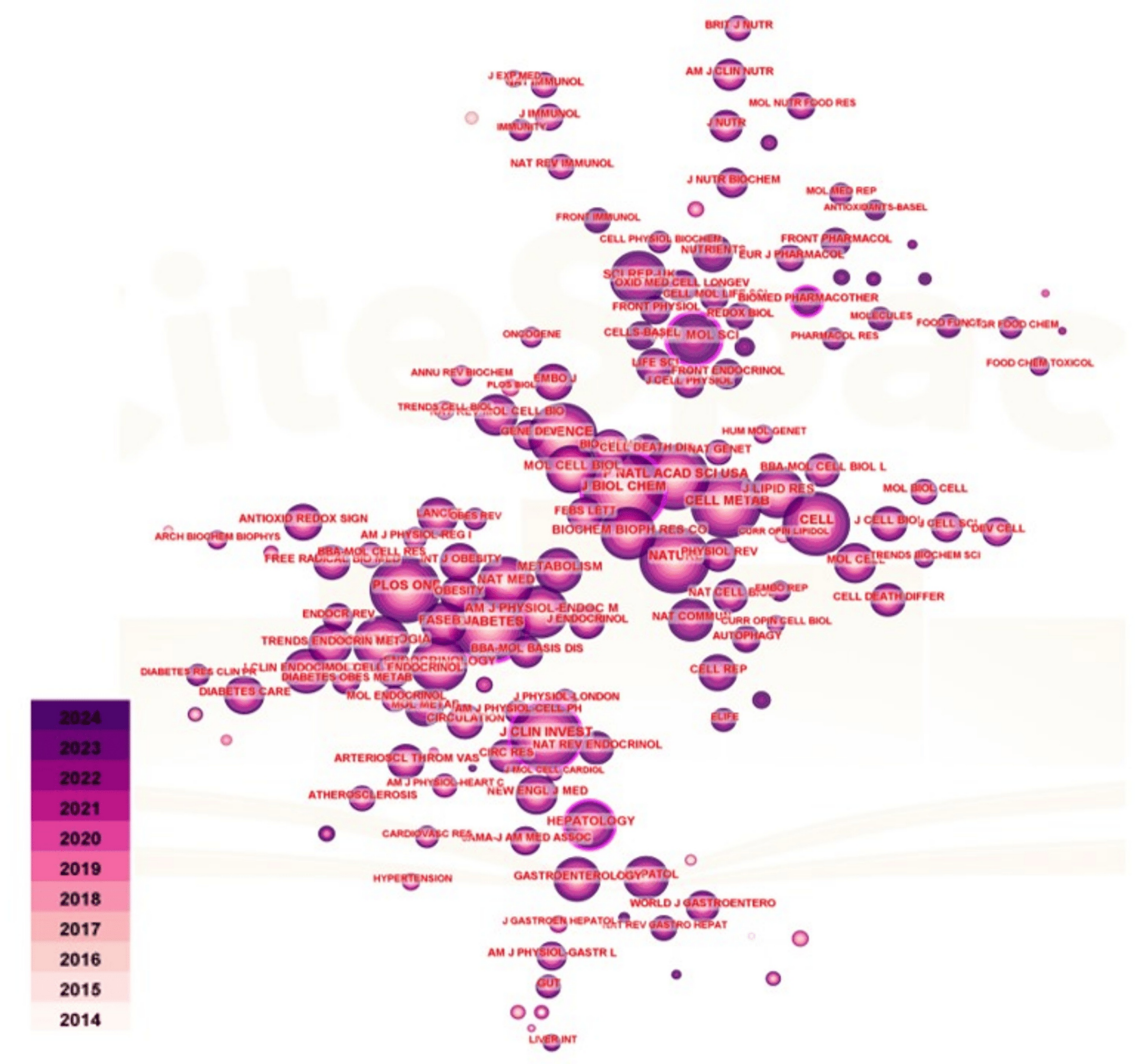
Co-citation visualization analysis in the field of obesity and endoplasmic reticulum stress research The size of the nodes represents the frequency of co-occurrence, and the gradient from beige (2014) to purple (2024) indicates different publication years. The connection lines between nodes represent the collaborative relationship, and their thickness indicates the intensity of the collaboration.

Figure 7 illustrates the journal co-citation network within the domain of obesity and ER stress research. The Journal of Biological Chemistry ranks first with 2,752 publications, followed by Diabetes (2,472 publications), PLOS One (2,436 publications), the Journal of Clinical Investigation (2,343 publications), and Nature (2,324 publications). The top five journals in terms of mediational centrality are the Journal of Biological Chemistry, Diabetes, the International Journal of Molecular Sciences, the Journal of Clinical Investigation, and Hepatology. These findings indicate that these journals play a pivotal role in facilitating research collaboration and advancing knowledge in the field of obesity and ER stress.

**Figure 8:**
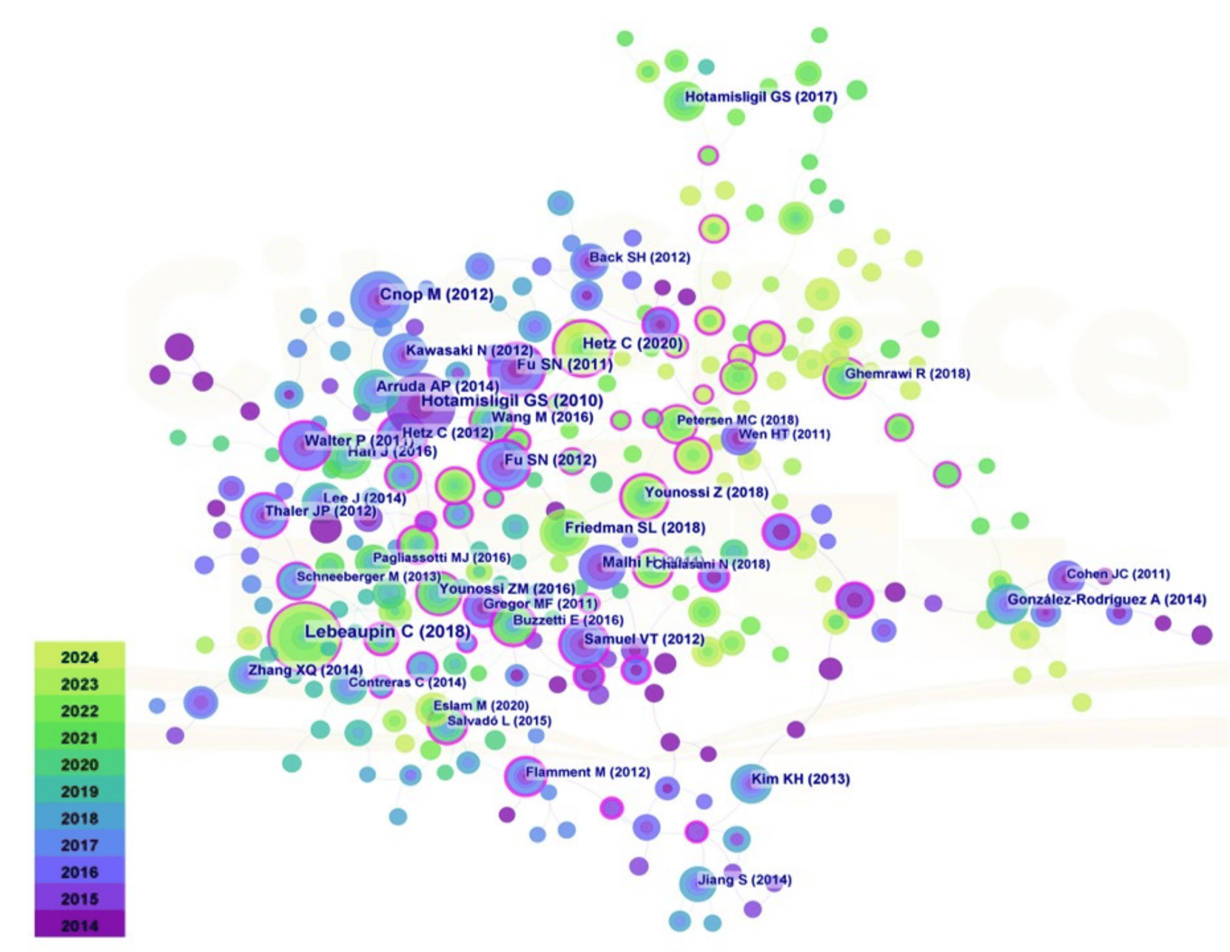
Visual analysis of references in the field of obesity and endoplasmic reticulum stress research The size of the nodes represents the frequency of co-occurrence, and the gradient from blue (2014) to green (2024) indicates different publication years. The connection lines between nodes represent the collaborative relationship, and their thickness indicates the intensity of the collaboration.

"Highly cited literature" refers to seminal or influential works within a field that have undergone rigorous peer review and are recognized for their high credibility and significant academic impact. Figure 8 illustrates the visual network of co-cited references in the research domain of obesity and ER stress. Notably, the most frequently cited paper was authored by Lebeaupin et al. in 2018, titled "Endoplasmic Reticulum Stress Signaling and the Pathogenesis of Non-Alcoholic Fatty Liver Disease," which has received 116 citations. This review examines the impact of ER stress on NAFLD in the context of obesity [17]. In patients with NAFLD, abnormal lipid accumulation in the liver disrupts protein homeostasis in hepatocytes, resulting in ER stress and the activation of the UPR pathway. This activation further regulates mechanisms related to lipid metabolism, insulin sensitivity, liver autophagy, calcium homeostasis, and hepatocyte apoptosis. The second most cited work, with 100 citations, is a 2010 publication by Hotamisligil et al., entitled "Endoplasmic Reticulum Stress and the Inflammatory Basis of Metabolic Disease." This comprehensive review elucidates the signaling pathways between the ER and the UPR, extending into the metabolic domain [18]. It highlights potential mechanisms of ER dysfunction in metabolic diseases. Given the ER's pivotal role in coordinating metabolic responses through its regulation of both synthetic and catabolic pathways for various nutrients, all three classical branches of the UPR are implicated in glucose metabolism [19]. Moreover, ER stress and UPR activate a spectrum of inflammatory and stress networks, including the JNK-AP1 and NF-κB-IKK pathways, as well as ROS and nitric oxide production [20-21]. Following this, Cnop et al.'s 2012 paper, "Endoplasmic Reticulum Stress, Obesity, and Diabetes," garnered 80 citations. This early systematic review delineates the mechanisms and roles of ER stress and UPR in obesity and type 2 diabetes [22]. Hypothalamic ER stress induces inflammation and contributes to resistance to leptin and insulin [23]. Hepatic ER stress results in steatosis and the progression of insulin resistance [24]. The UPR regulates hepatic lipid metabolism, triggers inflammation, and alters adipokine secretion, ultimately leading to ER stress in muscle tissue [25]. Ultimately, prolonged ER stress promotes insulin synthesis and contributes to the apoptosis of pancreatic β-cells. Collectively, these studies offer foundational insights into the mechanisms of ER stress in metabolic disorders such as obesity, enhancing our understanding of ER stress and its implications for disease onset and progression, as well as the maintenance of cellular metabolic activities and homeostasis.

**Figure 9:**
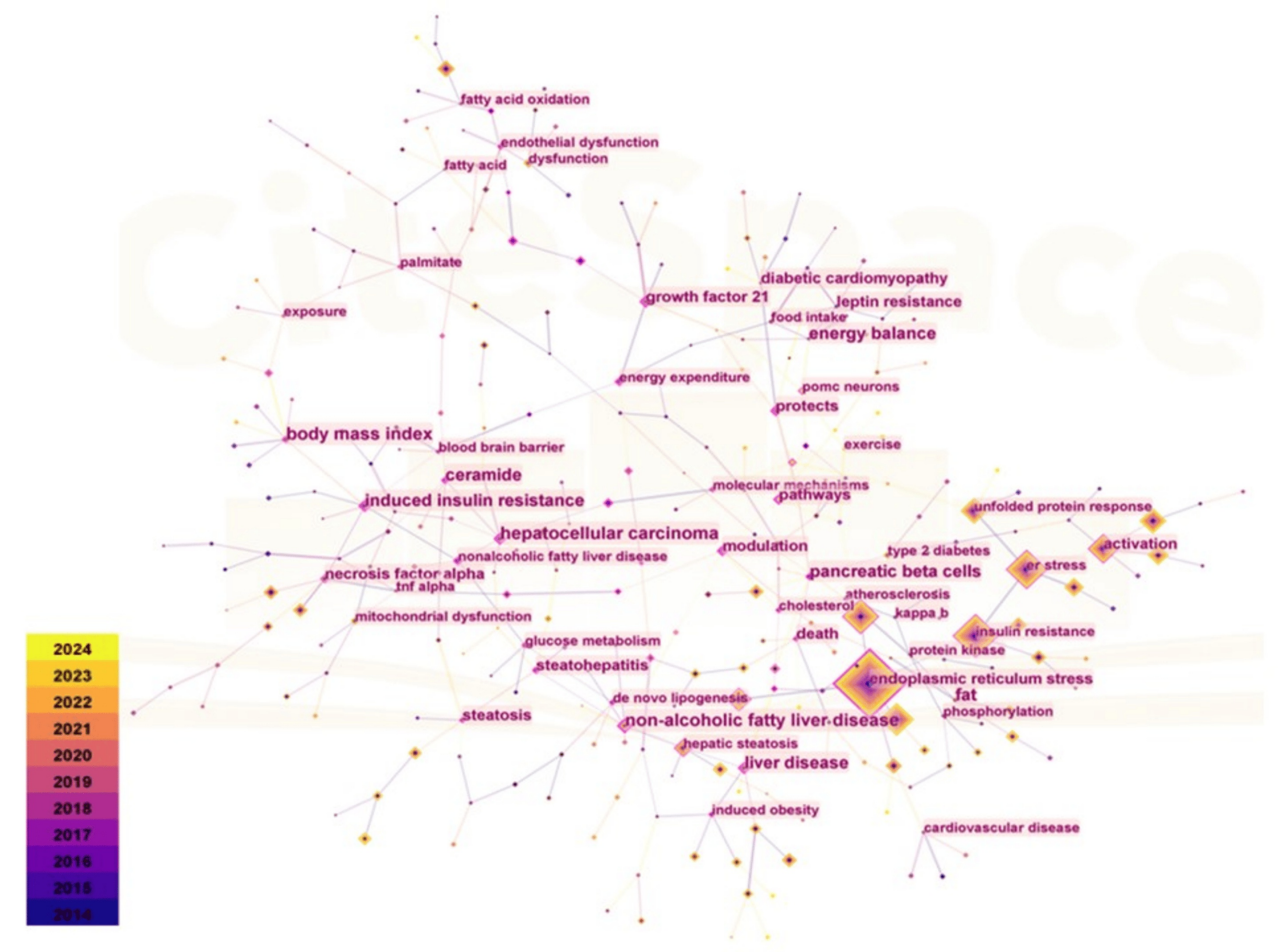
Analysis of high-frequency Keywords in the field of Obesity and Endoplasmic reticulum Stress research The size of the nodes represents the frequency of co-occurrence, and the gradient from purple (2014) to yellow (2024) indicates different publication years. The connecting lines between nodes represent the occurrence of a cooperative relationship.

Figure 9 presents the outcomes of a keyword co-occurrence study, which includes 292 nodes and 336 links. The high-frequency keywords related to obesity and ER stress highlight key research hotspots and frontier areas. Notably, "endoplasmic reticulum stress" ranks first with 2,596 occurrences. Other significant terms include insulin resistance (1,215 appearances), oxidative stress (853 appearances), obesity (736 appearances), and the UPR (557 appearances). In terms of intermediary centrality, "modulation" leads with a score of 0.48, followed by "pathways," "hepatocellular carcinoma," "induced insulin resistance," and "molecular mechanisms." This suggests that current research in this field is closely associated with insulin resistance and oxidative stress. 

**Figure 10:**
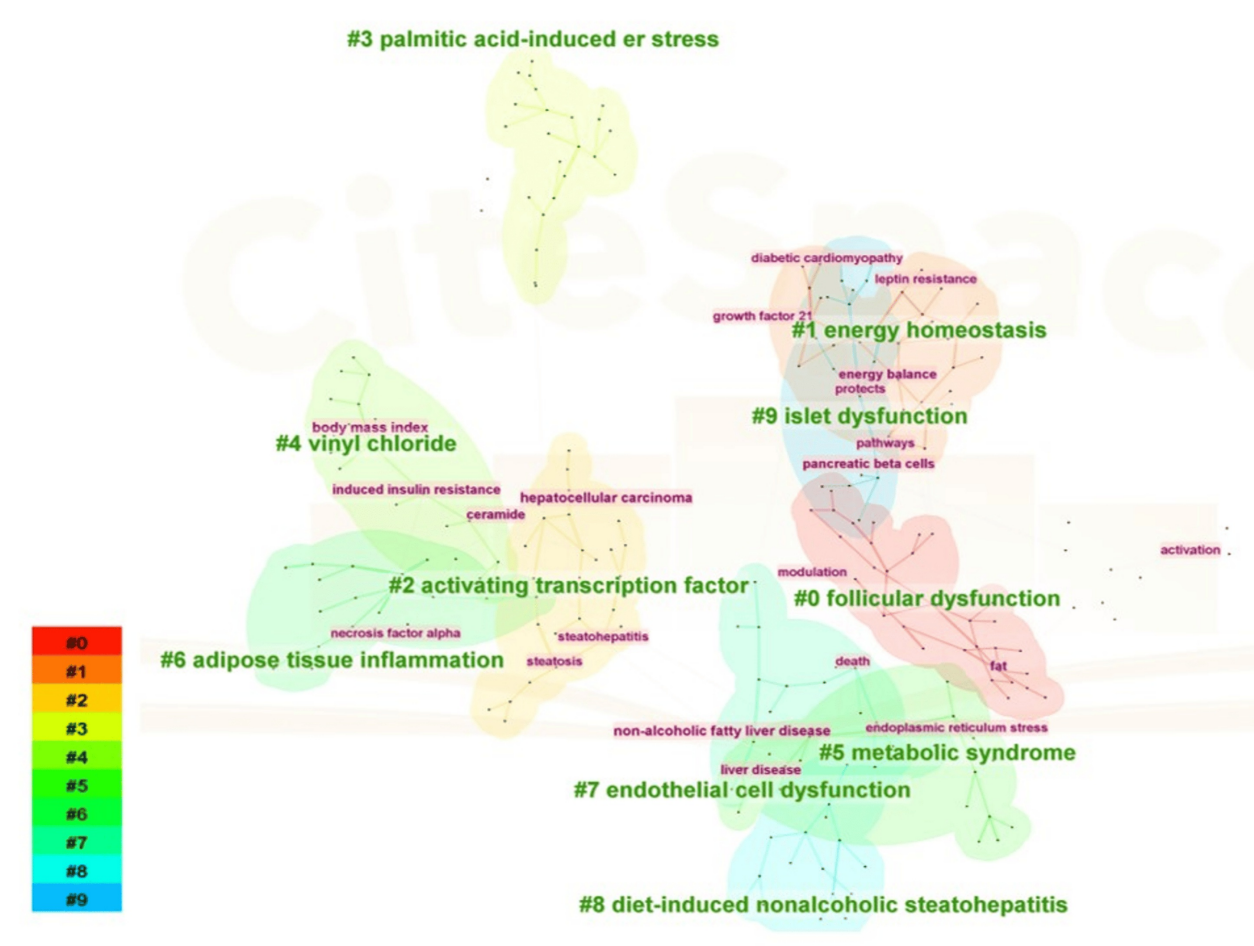
Keyword cluster analysis in the field of obesity and endoplasmic reticulum stress research. Different colors represent different clusters, ranging from #0 (blue) to #9 (red).

Figure 10 illustrates the cluster analysis of keywords associated with obesity and ER stress from 2014 to 2024. A total of 10 clusters were identified: #0 "follicular dysfunction," #1 "energy homeostasis," #2 "activating transcription factor," #3 "palmitic acid-induced ER stress," #4 "vinyl chloride," #5 "metabolic syndrome," #6 "adipose tissue inflammation," #7 "endothelial cell dysfunction," #8 "diet-induced nonalcoholic steatohepatitis," and #9 "islet dysfunction." These clusters primarily emphasize diseases such as follicular dysfunction and metabolic syndrome, as well as pathophysiological mechanisms, including energy metabolism, ER stress, and inflammatory responses. Studies have demonstrated that local factors within the ovary and gonadotropin collaborate to regulate the follicle microenvironment. The physiological role of ER stress and the UPR in the ovary remains unclear.

**Figure 11:**
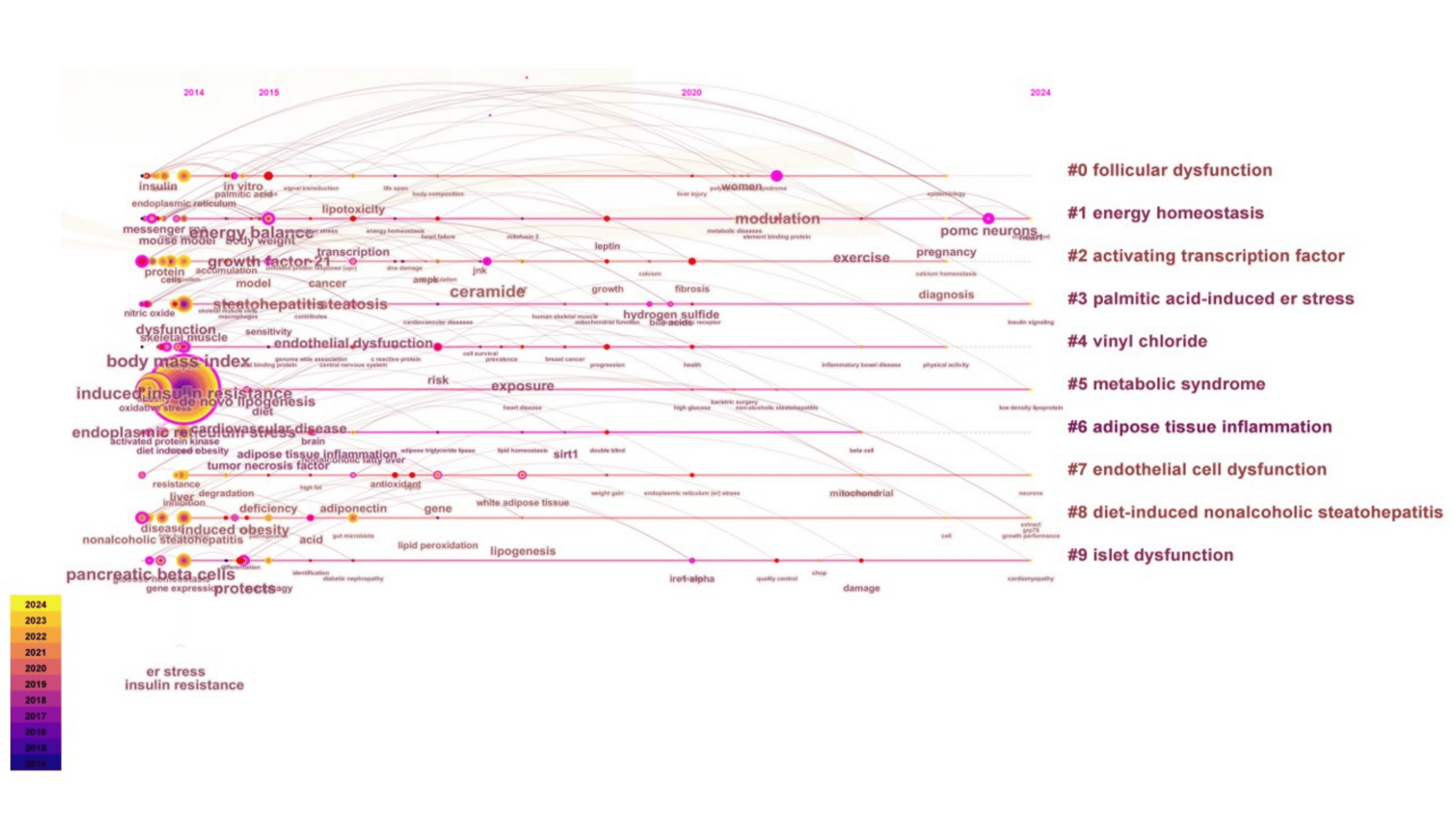
Keyword clustering timeline analysis in the field of obesity and endoplasmic reticulum stress research The horizontal axis from left to right represents the time axis from 2014 to 2024. The size of the nodes indicates the collinear frequency of the keywords, and the color gradient from purple (2014) to yellow (2024) represents the publication year. The connecting lines represent the occurrence of collaborative connections.

Figure 11 illustrates the visualization of the superposition of the keyword cluster time evolution. Throughout the entire data acquisition period, key terms such as "energy homeostasis," "palmitic acid-induced stress," "metabolic syndrome," "endothelial cell dysfunction," and "diet-induced nonalcoholic steatohepatitis" are consistently present. Notably, "insulin resistance" in the context of metabolic syndrome has consistently garnered significant research attention and appears most frequently. This figure facilitates the analysis of stage-specific hotspots and developmental trajectories of key terms in obesity and ER stress research.

**Figure 12:**
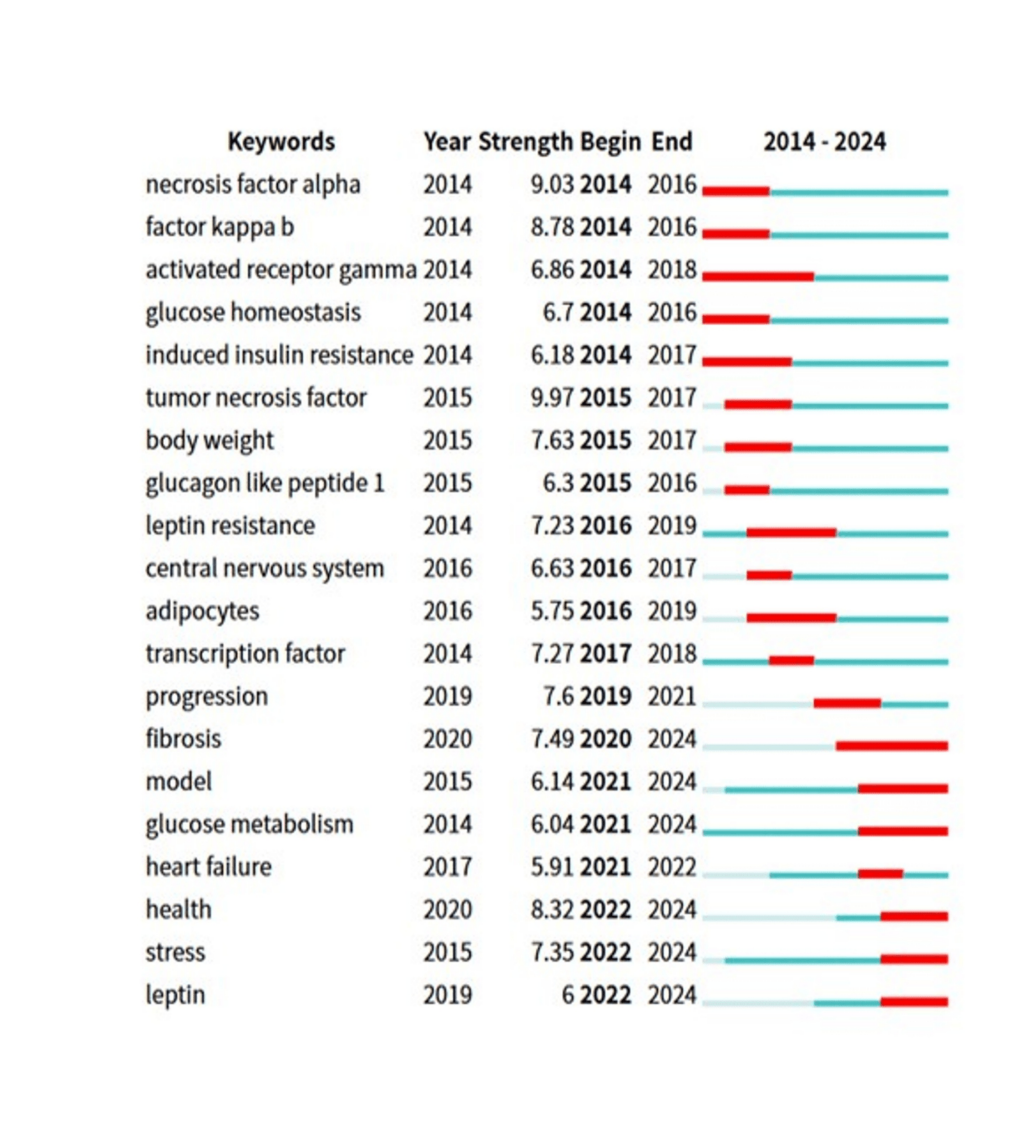
Citation analysis of the top 20 keywords in the field of obesity and endoplasmic reticulum stress research Obvious citation surges are represented by the red sections on the horizontal axis, indicating that the keyword has received a large number of citations during that time period, to clarify the explosive trends of different keywords over time.

The sudden increase in keyword frequency indicates a significant rise in research interest over a specific period, potentially guiding future research directions. Figure 12 illustrates the top 20 keywords with the strongest citation bursts in studies related to obesity and ER stress. Between 2014 and 2016, the burst intensity of tumor necrosis factor alpha was the highest at 9.03. From 2016 to 2018, the highest burst intensity was observed for activated receptor gamma at 6.86. During the period from 2019 to 2021, the term "progression" exhibited the highest burst intensity at 7.6. From 2021 to 2024, fibrosis demonstrated the highest burst intensity at 7.49.

Further review how obesity causes ER stress in the body and thereby affects metabolic, inflammatory, and other pathways. Obese patients exhibit elevated levels of free fatty acids (FFA), which have been shown to induce ER stress and contribute to lipotoxicity-induced beta cell death [26]. Cholesterol transport to the ER membrane leads to depletion of Ca2+ storage, thereby activating the UPR, including the IRE1α-XBP1 pathway [27]. Under ER stress, IRE1α undergoes dimerization and trans-autophosphorylation, which leads to the allosteric activation of its C-terminal RNase domain. This catalyzes the unconventional splicing of the mRNA encoding XBP1, producing spliced Xbp1 mRNA [28]. This mRNA encodes a more stable and transcriptionally active XBP1s protein, which initiates the major UPR pathway by upregulating protein chaperones, ERAD components, and molecules involved in ER biogenesis [29].

In cultured insulinoma-β cell lines and primary islets, high glucose stimulates the phosphorylation of IRE1α at Ser724 in its kinase activation domain [30], leading to increased splicing of Xbp1 mRNA without inducing activation of the PERK-eIF2α branch of UPR [31]. Although the molecular signaling mechanism through which the IRE1α arm is selectively activated by glucose remains largely unknown, this glucose-responsive phosphorylation of IRE1α indicates its association with insulin biosynthesis. IRE1α is activated by both anabolic and catabolic hormones. In mice on a high-fat diet, insulin resistance is linked to hyperinsulinemia, which is characterized by increased Ser724 phosphorylation of liver IRE1α and splicing of Xbp1 mRNA [32].

IRE1α interacts with TNF receptor-associated factor 2 and is linked to the JNK and IKKβ-NF-κB inflammatory pathways [33]. IRE1α may play a crucial role in detecting cytoplasmic danger signals that initiate pro-inflammatory responses, such as IL-6 production, during tissue damage or microbial infection [34]. These findings suggest that IRE1α plays a crucial role in linking ER stress to inflammation, which is particularly relevant in the context of obesity-related metabolic inflammation.

## Conclusions

This study provides a comprehensive analysis of the molecular mechanisms underlying obesity and ER stress, utilizing bibliometric and visualization techniques to identify key research trends and collaborative networks. The findings highlight the critical role of ER stress in metabolic syndromes, including obesity, by demonstrating its effects on reducing lipid accumulation and inflammation, enhancing insulin sensitivity, and improving cardiovascular function. In terms of research methodologies, investigations into ER stress-related signaling pathways, such as the UPR, ERAD, and autophagy, have continued to advance. The mechanisms by which these pathways respond to ER stress, maintain cellular homeostasis, and contribute to the development of obesity-related diseases are gradually being clarified. Regarding research trends, the focus has shifted from exploring fundamental mechanisms to identifying potential therapeutic targets, such as modulating ER stress response pathways to alleviate cellular stress and improve disease outcomes. As related research progresses, the specific effects and precise regulatory mechanisms of ER stress in various diseases are expected to become central themes for future investigation.

## References

[1] Rhee EJ (2022). The influence of obesity and metabolic health on vascular health. Endocrinol Metab (Seoul).

[2] Du H, Ren X, Bai J, Yang W, Gao Y, Yan S (2022). Research progress of ferroptosis in adiposity-based chronic disease (ABCD). Oxid Med Cell Longev.

[3] Ajoolabady A, Liu S, Klionsky DJ (2022). ER stress in obesity pathogenesis and management. Trends Pharmacol Sci.

[4] Donahue E, Hepowit NL, Keuchel B (2024). ER-phagy drives age-onset remodeling of endoplasmic reticulum structure-function and lifespan (Preprint). bioRxiv.

[5] Wiseman RL, Mesgarzadeh JS, Hendershot LM (2022). Reshaping endoplasmic reticulum quality control through the unfolded protein response. Mol Cell.

[6] Ma K, Zhang Y, Zhao J, Zhou L, Li M (2024). Endoplasmic reticulum stress: bridging inflammation and obesity-associated adipose tissue. Front Immunol.

[7] Lemmer IL, Willemsen N, Hilal N, Bartelt A (2021). A guide to understanding endoplasmic reticulum stress in metabolic disorders. Mol Metab.

[8] Vallée D, Blanc M, Lebeaupin C, Bailly-Maitre B (2020). Endoplasmic reticulum stress response and pathogenesis of non-alcoholic steatohepatitis (Article in French). Med Sci (Paris).

[9] Kim H, Lee DS, An TH, Park HJ, Kim WK, Bae KH, Oh KJ (2021). Metabolic spectrum of liver failure in type 2 diabetes and obesity: From NAFLD to NASH to HCC. Int J Mol Sci.

[10] Ye Z, Liu G, Guo J, Su Z (2018). Hypothalamic endoplasmic reticulum stress as a key mediator of obesity-induced leptin resistance. Obes Rev.

[11] Kang Z, Chen F, Wu W, Liu R, Chen T, Xu F (2022). UPR(mt) and coordinated UPR(ER) in type 2 diabetes. Front Cell Dev Biol.

[12] Yilmaz E (2017). Endoplasmic reticulum stress and obesity. Adv Exp Med Biol.

[13] Ajoolabady A, Lebeaupin C, Wu NN, Kaufman RJ, Ren J (2023). ER stress and inflammation crosstalk in obesity. Med Res Rev.

[14] Congur I, Mingrone G, Guan K (2025). Targeting endoplasmic reticulum stress as a potential therapeutic strategy for diabetic cardiomyopathy. Metabolism.

[15] Sugimoto CR, Ahn YY, Smith E, Macaluso B, Larivière V (2019). Factors affecting sex-related reporting in medical research: a cross-disciplinary bibliometric analysis. Lancet.

[16] Chen C, Hu Z, Liu S, Tseng H (2012). Emerging trends in regenerative medicine: a scientometric analysis in CiteSpace. Expert Opin Biol Ther.

[17] Lebeaupin C, Vallée D, Hazari Y, Hetz C, Chevet E, Bailly-Maitre B (2018). Endoplasmic reticulum stress signalling and the pathogenesis of non-alcoholic fatty liver disease. J Hepatol.

[18] Hotamisligil GS (2010). Endoplasmic reticulum stress and the inflammatory basis of metabolic disease. Cell.

[19] Liu Y, Yang X, Zhou J (2024). OSGEP regulates islet β-cell function by modulating proinsulin translation and maintaining ER stress homeostasis in mice. Nat Commun.

[20] Gulzar F, Chhikara N, Kumar P, Ahmad S, Yadav S, Gayen JR, Tamrakar AK (2024). ER stress aggravates NOD1-mediated inflammatory response leading to impaired nutrient metabolism in hepatoma cells. Biochem Biophys Res Commun.

[21] Shi C, Zhang Q, Li Y, Zhao J, Wang C, Zhang Y (2024). Polyethylene glycol loxenatide protects diabetic kidneys by inhibiting GRP78/PERK/eIF2α pathway, and improves cardiac injury by suppressing TLR4/NF-κB inflammatory pathway. BMC Cardiovasc Disord.

[22] Cnop M, Foufelle F, Velloso LA (2012). Endoplasmic reticulum stress, obesity and diabetes. Trends Mol Med.

[23] Zhang S, Chen Y, Yang R (2025). Aerobic exercise improves energy and glucose homeostasis through hypothalamic Mitofusion 2-rescued endoplasmic reticulum stress in diet-induced obese mice. Diabetes Obes Metab.

[24] Khoi CS, Lin TY, Chiang CK (2024). Targeting insulin resistance, reactive oxygen species, inflammation, programmed cell death, ER stress, and mitochondrial dysfunction for the therapeutic prevention of free fatty acid-induced vascular endothelial lipotoxicity. Antioxidants (Basel).

[25] Salagre D, Navarro-Alarcón M, González LG, Elrayess MA, Villalón-Mir M, Haro-López R, Agil A (2024). Melatonin ameliorates organellar calcium homeostasis, improving endoplasmic reticulum stress-mediated apoptosis in the vastus lateralis muscle of both sexes of obese diabetic rats. Antioxidants (Basel).

[26] Yin ZY, He SM, Zhang XY (2025). Apolipoprotein B100 acts as a tumor suppressor in ovarian cancer via lipid/ER stress axis-induced blockade of autophagy. Acta Pharmacol Sin.

[27] Feng B, Yao PM, Li Y (2003). The endoplasmic reticulum is the site of cholesterol-induced cytotoxicity in macrophages. Nat Cell Biol.

[28] Fu F, Doroudgar S (2022). IRE1/XBP1 and endoplasmic reticulum signaling - from basic to translational research for cardiovascular disease. Curr Opin Physiol.

[29] Walter P, Ron D (2011). The unfolded protein response: from stress pathway to homeostatic regulation. Science.

[30] Hassler JR, Scheuner DL, Wang S (2015). The IRE1α/XBP1s pathway is essential for the glucose response and protection of β cells. PLoS Biol.

[31] Qiu Y, Mao T, Zhang Y (2010). A crucial role for RACK1 in the regulation of glucose-stimulated IRE1alpha activation in pancreatic beta cells. Sci Signal.

[32] Ning J, Hong T, Ward A, Pi J, Liu Z, Liu HY, Cao W (2011). Constitutive role for IRE1α-XBP1 signaling pathway in the insulin-mediated hepatic lipogenic program. Endocrinology.

[33] Madhavan A, Kok BP, Rius B (2022). Pharmacologic IRE1/XBP1s activation promotes systemic adaptive remodeling in obesity. Nat Commun.

[34] Kim G, Lee J, Ha J, Kang I, Choe W (2023). Endoplasmic reticulum stress and its impact on adipogenesis: molecular mechanisms implicated. Nutrients.

